# A new method for determination of varicella-zoster virus immunoglobulin G avidity in serum and cerebrospinal fluid

**DOI:** 10.1186/1471-2334-4-33

**Published:** 2004-09-08

**Authors:** Ralf-Herbert Kneitz, Jörg Schubert, Franz Tollmann, Wolfgang Zens, Klaus Hedman, Benedikt Weissbrich

**Affiliations:** 1 Institute of Virology and Immunobiology, University of Würzburg, Versbacher Str. 7, 97078 Würzburg, Germany; 2 Dade Behring Marburg GmbH, P. O. Box 11 49, 35001 Marburg, Germany; 3 Haartman Institute, Department of Virology, University of Helsinki and Helsinki University Central Hospital (HUCH), FIN-00290, Helsinki, Finland

## Abstract

**Background:**

Avidity determination of antigen-specific immunoglobulin G (IgG) antibodies is an established serological method to differentiate acute from past infections. In order to compare the avidity of varicella-zoster virus (VZV) IgG in pairs of serum and cerebrospinal fluid (CSF) samples, we developed a new technique of avidity testing, the results of which are not influenced by the concentration of specific IgG.

**Methods:**

The modifications introduced for the new VZV IgG avidity method included the use of urea hydrogen peroxide as denaturing reagent, the adaptation of the assay parameters in order to increase the sensitivity for the detection of low-level VZV IgG in CSF, and the use of a new calculation method for avidity results. The calculation method is based on the observation that the relationship between the absorbance values of the enzyme immunoassays with and without denaturing washing step is linear. From this relationship, a virtual absorbance ratio can be calculated. To evaluate the new method, a panel of serum samples from patients with acute and past VZV infection was tested as well as pairs of serum and CSF.

**Results:**

For the serum panel, avidity determination with the modified assay gave results comparable to standard avidity methods. Based on the coefficient of variation, the new calculation method was superior to established methods of avidity calculation.

**Conclusions:**

The new avidity method permits a meaningful comparison of VZV IgG avidity in serum and CSF and should be of general applicability for easy determination of avidity results, which are not affected by the concentration of specific IgG.

## Background

In addition to the determination of immunoglobulin M (IgM) antibodies, the avidity of immunoglobulin G (IgG) antibodies is an important parameter for the diagnosis or exclusion of acute infections. Testing of IgG avidity has been applied for a large variety of pathogens (reviewed in [1,2]). A correct diagnosis is especially important for infections during pregnancy with rubella virus, cytomegalovirus and *Toxoplasma gondii*. Therefore, avidity testing has been particularly useful for these pathogens.

Determination of antibody avidity is usually based on the separation of low and high avidity antibodies by denaturing agents in enzyme immunoassays (EIA) or immunofluorescence assays. Several agents such as guanidine hydrochloride [3], diethylamine [4], thiocyanate [5] or urea [6] have been used for this purpose. These protein denaturants have been either included in the sample diluent (diluting principle) or in the washing buffer after the serum incubation step (eluting principle). Calculation of the avidity result has been performed in numerous ways. Mostly, avidity is expressed as percent ratio of antibody titers or EIA absorbance values with and without denaturation. Avidity results based on end-point titration with and without denaturant are considered to be the gold standard for avidity determination [7]. This technique has an excellent sensitivity and specificity and is considered not to be influenced by the concentration of the specific IgG. To reduce the considerable expense and labour required for the titration curves, simplified avidity tests based on single-point determinations using EIAs have been described [6,8-11]. In these assays, EIA absorbance values or antibody titers mathematically derived from single dilutions have been used for avidity calculation. The avidity results based on single-point absorbance values are to some extent influenced by the concentration of specific IgG, but this is usually not critical for the distinction between low and high avidity.

Diagnosis or exclusion of acute infection is the most common but not the only application of avidity determination. Determination of antibody avidity in pairs of serum and cerebrospinal fluid (CSF) samples has been suggested as a diagnostic means to detect intrathecal antibody synthesis [12] and to differentiate viral encephalitis from multiple sclerosis [13]. Longitudinal measurements of HIV avidity have been proposed to be useful for the assessment of HIV progression [14]. Because both of these applications involve comparative measurements of avidity where small differences may be of diagnostic importance, a technique for accurate avidity determination is required which is independent of antibody concentrations.

The aim of our study was to establish an avidity assay that can be used for comparison of the avidities of varicella-zoster virus (VZV) IgG in serum and CSF. In order to achieve this aim, several modifications of a commercial VZV IgG assay were introduced. These modifications include the use of a novel denaturing agent and a new method to calculate the avidity results.

## Methods

### Serum and CSF samples

The serum and CSF samples used in this study had been sent to the virological laboratory at the University of Würzburg for routine VZV testing and were stored in aliquots at -20°C. Two groups of serum samples were analyzed for the evaluation of the standard and modified VZV avidity assay. Group 1 consisted of 28 samples from 19 patients with acute or recent VZV infection and included 5 follow-up samples of patient F19, spanning a period of 11 months. The cases of this group fulfilled the following criteria: the presence of clinical symptoms suggestive of varicella, information on the disease onset, and a positive VZV IgM (Enzygnost Anti-VZV/IgM, Dade Behring, Marburg, Germany). Group 2 consisted of 37 samples from 37 subjects with infection in the distant past. In the subjects of this group, VZV IgG antibodies had been detected in previous samples taken at least 8 months earlier. For initial evaluation of the VZV avidity assay for CSF samples, three sample pairs of serum and CSF were tested, two from patients with VZV encephalitis (V2.1 and V9.2) and one from a patient with multiple sclerosis (M22).

### Standard VZV IgG avidity determination

VZV IgG avidity determination of serum samples was performed in a semi-automated fashion using the Enzygnost Anti-VZV/IgG test kit (Dade Behring) with some modifications. Each of the sera diluted 1:231 in sample buffer was placed in two antigen coated microtiter plate wells. After 60 min at 37°C, one well was washed according to the instructions with the supplied washing buffer. The other well was washed with a solution of urea, or urea hydrogen peroxide, in the supplied washing buffer (for details, see Results) and once with the washing buffer only. The subsequent steps were carried out according to the instructions in an automated fashion using the Behring Elisa Processor III (BEP III; Dade Behring). Peroxidase-conjugated anti-human IgG was added in a 1:50 dilution and the plate was incubated at 37°C for 60 min. After three further washing steps with the supplied washing buffer, tetramethylene benzidine dihydrochloride was added as substrate and kept at room temperature for 30 min. The reaction was stopped with 0.5 N sulfuric acid. Control samples of acute and past VZV infections were included in each run. Where appropriate, VZV IgG antibodies were quantified by an one-point-quantification method (α-method, Dade Behring) according to the instructions of the manufacturer.

### VZV IgG avidity determination of CSF-serum-pairs

To increase the sensitivity of the VZV IgG EIA in order to detect low-titer VZV IgG antibodies in CSF, the standard serum assay was modified as follows. The sample incubation time was increased to 180 min; the anti-human IgG was used in a 1:30 dilution and its incubation time was increased to 90 min. All incubations for the CSF assay were performed at room temperature because variation was found to be lower compared to incubation at 37°C. For the serum samples tested for evaluation purposes, the standard dilution of 1:231 was increased by a factor of 6 to yield a final dilution of 1:1386.

Determination of VZV IgG avidity in CSF was always done in parallel with serum samples from the same time-point. Both serum and CSF were tested in at least four dilutions of a two-fold titration series. The starting dilution for each sample was derived from routine determinations of VZV IgG titers. For CSF samples, the starting dilution was at least 1:6.

### Calculation of the avidity index

VZV IgG avidity was calculated by various methods. First, it was calculated as the ratio of EIA absorbance values obtained with and without the denaturing washing step. Alternatively, one-point quantification titers (see above) instead of absorbance values were used for avidity calculation.

Two additional methods were used for determination of VZV IgG avidity in CSF and the corresponding serum samples. One method involved the use of the software "Avidity 1.2", based on curve-fitting analysis of serial dilutions [15]. Secondly, we developed a new calculation method based on our observation from dilution series that the relationship between the absorbance values obtained without and with denaturing agents is linear. Thus, the relationship can be described by the equation y = m × x + c, where m is the slope, c the intercept, x the absorbance without the denaturant (abs_ref_) and y the absorbance with the denaturant (abs_denat_). The m- and c-values of the equation for each sample were calculated with the software Excel (Microsoft) from two or more experimentally derived data points. It is essential, that the absorbance values obtained without denaturant (abs_ref_) fall in the linear range of the enzyme immunoassay. For the VZV IgG assay with modified assay conditions, the linear range of the absorbance values without denturant extended from approximately 0.200 to 2.800. Avidity was determined from the linear equation for various virtual x-values as the percent ratio of the absorbance values with and without denaturing agent, i. e. avidity = (abs_denat_/abs_ref_) = (m × x + c)/x. The result was referred to as virtual absorbance ratio.

## Results

**Figure 1:**
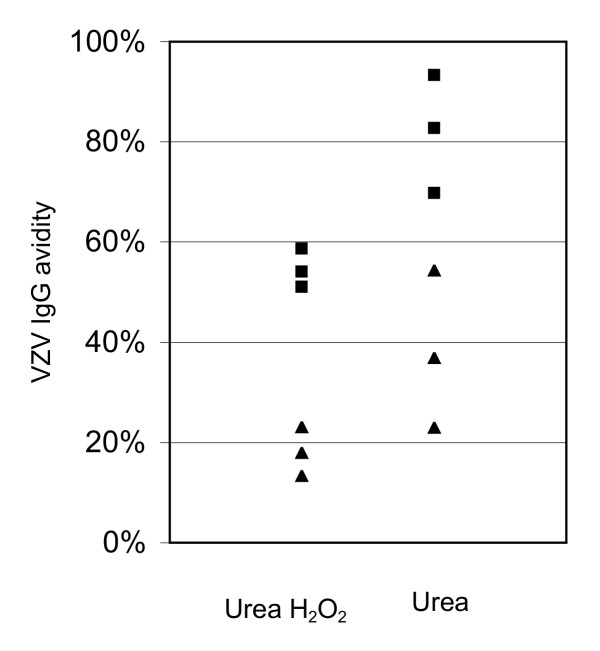
Optimization of the denaturing washing conditions. VZV IgG avidity of a panel of serum samples from patients with acute (▲) and past (■) VZV infection was tested with urea hydrogen peroxide (H_2_O_2_) and urea as denaturing agent.

In order to evaluate the usefulness of the Enzygnost VZV IgG EIA for avidity testing, a panel of serum samples from patients with acute VZV infection of defined onset (group 1) and from controls with VZV infection in the distant past (group 2) was tested. In a preliminary experiment with three samples from each group, two different denaturing conditions were employed. The separation of the two sample groups with one 3 min washing step using 4 M urea hydrogen peroxide was superior to three 5 min washing steps with 5 M urea (Table 1, Figure 1). Therefore, urea hydrogen peroxide was used for the following experiments.

**Figure 2:**
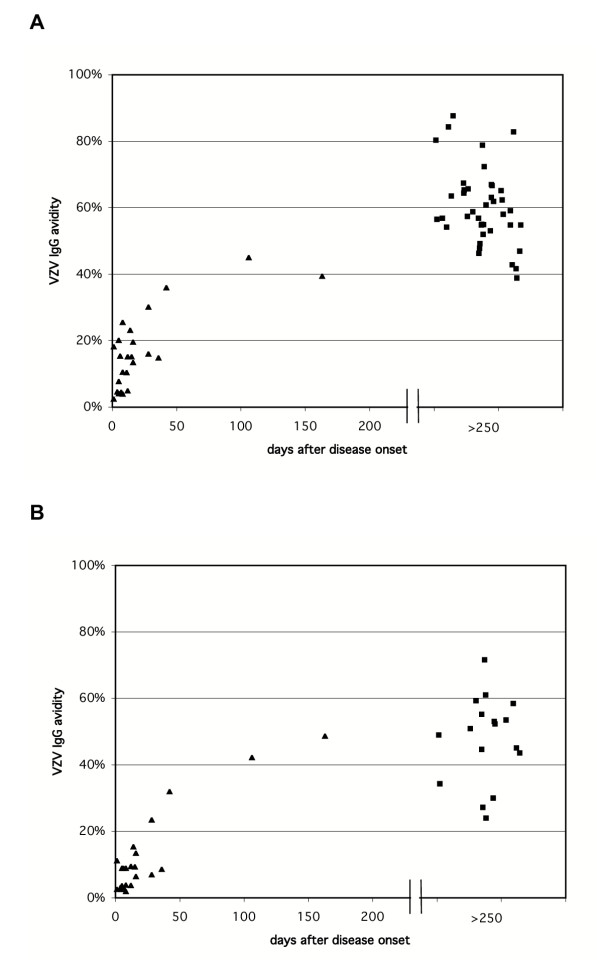
Evaluation of different VZV IgG avidity assays with serum samples from patients with acute (▲) and past (■) VZV infection. The Enzygnost Anti-VZV/IgG test kit was used with standard conjugate concentration and standard incubation times (A) and with modified assay conditions (B; see text).

The results of avidity testing with urea hydrogen peroxide of all serum samples of group 1 and 2 are shown in Figure 2a. When avidity indices were calculated as the percent ratio of absorbance values, there was a clear distinction of the avidity values from both groups. Using a cut-off of 37 %, all samples from patients with disease onset of less than 50 days had avidity values below the cut-off, while all samples from the control group with VZV infection in the distant past had avidity values above the cut-off. Thus, a cut-off of 37 % resulted in a sensitivity and specificity of 100 % for the detection and exclusion of VZV infections within in the last 50 days.

The standard result format of the Enzygnost VZV IgG assay are titers that are mathematically derived from single-point absorbance values (one-point-quantification). Because it had been shown previously for the Epstein-Barr virus (EBV) assay that avidity index calculations based on one-point-quantification titers gave results better than calculations using absorbance values [11], the avidity indices were recalculated using VZV IgG one-point-quantification titers instead of absorbance values. There was an excellent correlation between both methods of avidity index calculation, but in contrast to the EBV assay, the one-point-quantification method was not superior to that using absorbance values (data not shown).

In general, IgG concentrations in CSF are by a factor of 1:200 to 1:1000 lower than in the corresponding serum. Therefore, the detection sensitivity of the VZV IgG assay was increased by prolonging incubations times and increasing the concentration of the anti-human IgG conjugate in order to reliably detect VZV IgG in all CSF samples from patients with positive serum VZV IgG. To study the effect of these assay modifications on avidity determination, all serum samples of group 1 and 17 randomly chosen samples of group 2 were retested with the modified assay. All the serum samples were tested in dilution 1:1386 instead of 1:231 used in the standard serum assay. The results are shown in Figure 2b. An avidity cut-off of 23 % resulted in a sensitivity of 95 % and specificity of 95 % for the detection and exclusion of VZV infections within the last 50 days, respectively. Although the standard serum conditions gave better results, there was again a clear separation between samples from both groups. Based on these results, the avidity assay with urea hydrogen peroxide and modified assay conditions to increase the sensitivity for VZV IgG detection was considered appropriate for use in VZV IgG avidity studies with CSF and is henceforth called the *CSF avidity assay*.

**Figure 3:**
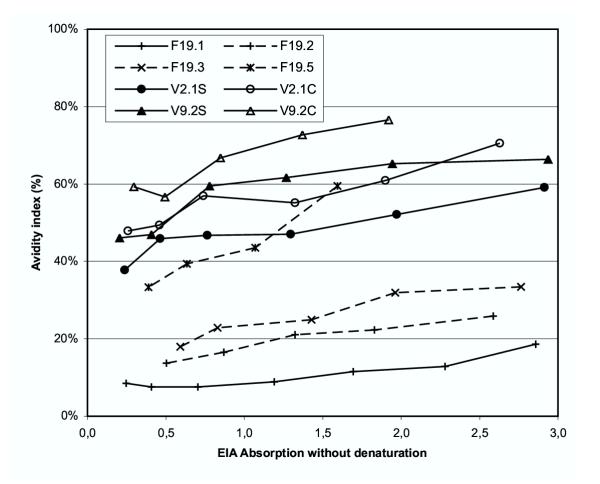
Influence of the concentration of specific IgG on single-point avidity indices. Two-fold dilution series of serum and CSF samples were tested in the CSF avidity assay and the avidity index was calculated as the percent ratios of absorbance values for each dilution. F19: Follow-up samples of a patient with acute VZV infection. V2.1 and V9.2: Pairs of serum (S) and CSF (C) of a patient with VZV encephalitis.

For meaningful interpretation of avidity values in CSF, it is necessary to test serum samples obtained in parallel with the CSF. Because of the large difference of the IgG concentrations between serum and CSF, comparison of the avidity results necessitates independence of IgG concentration. To study this issue, CSF and serum samples were tested in twofold dilution series with the CSF avidity assay. Avidity was calculated as percent ratio of absorbance values from each dilution. The results for representative CSF and serum samples are shown in Figure 3. The avidity indices of most of the samples varied considerably depending on the sample dilution. For some samples, the range of avidity indices was greater than 20 %. The coefficient of variation (CV) was mostly higher than 10 % (examples in Table 2). Thus, this method could not be used for comparative testing of serum and CSF samples. Alternatively, avidity indices were calculated with the software "Avidity 1.2", which is based on a mathematical model described previously by Korhonen et al. [15]. The software calculates avidity values from two sample dilutions, each tested with and without protein denaturant. To evaluate the influence of the working dilutions on the avidity indices obtained with this software, the avidity indices were calculated from different pairs of dilutions of the same sample. The results for representative examples are shown in Table 3. The CV ranged from 5.9 % to 19.7 %.

**Figure 4:**
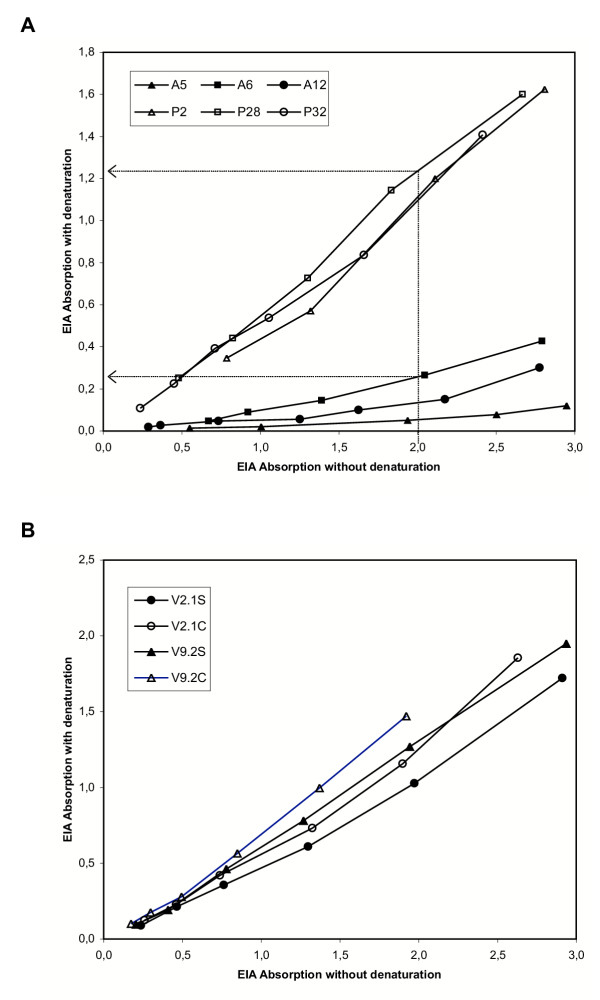
Linear relationship between absorbance values without and with denaturing washing step. Absorption-absorption-diagrams of dilution series of samples from (A) patients with acute (closed symbols) and past VZV infection (open symbols) and (B) of pairs of serum (closed symbols) and CSF samples (open symbols). For two examples, the determination of y-values for the virtual x-value 2.0 is represented graphically (dotted lines with arrow heads). Avidity is calculated as the ratio of absorbance y divided by absorbance x.

**Figure 5:**
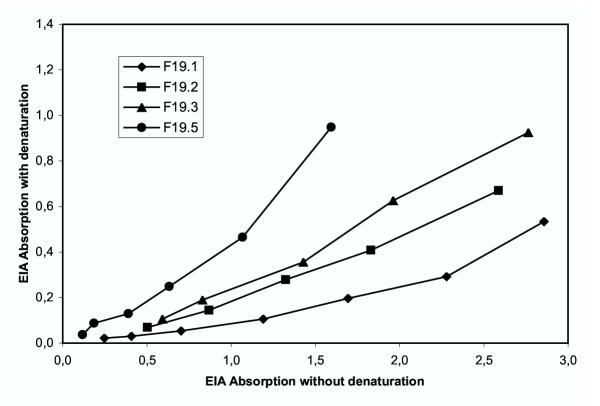
Absorption-absorption-diagram of follow-up serum samples of patient (F19) with acute VZV infection tested in the CSF VZV IgG avidity assay. F19.1/2/3/5: 15/28/42/336 days after onset of disease, respectively.

Because the two methods for avidity calculation described above were significantly influenced by the chosen working dilutions, we developed a new calculation method of antibody avidity. It is based on our observation that the relationship between absorbance values without and with denaturing agent is nearly linear. This relationship can therefore be described by the linear equation y = m × x + c, where m is the slope, c the intercept, x the absorbance without denaturant and y the absorbance with denaturant. Representative examples from patients with acute and past VZV infection and of serum and CSF pairs are shown in Figure 4. After experimental determination of the sample-specific m- and c-values, this observation allows calculating the y-value for any virtual absorbance without denaturation (x-value). Absorbance results from two dilutions are required in order to calculate the m- and c-values for a sample. In order to evaluate the influence of the chosen sample dilution on the avidity index derived from the virtual absorbance ratio, several samples were tested in series of twofold dilutions with four or more steps. The m- and c-values were calculated from different pairs of dilution steps for each sample. As a result of preliminary experiments, the absorbance 2.0 was chosen as the virtual x-value for the calculation of avidity values with the new method. (The linear range of the absorbances in the CSF assay format extended up to 2.8.) Table 3 shows that the CV for the avidity indices calculated by the new method from different pairs of dilution steps was lower than the CV obtained with the software "Avidity 1.2" from the same dilution series. Based on theoretical considerations we speculated that the slope of the linear equation by itself might represent the avidity of a sample, i.e. the steeper the linear curve, the higher the avidity. This was in agreement with the data from follow-up samples of a patient with acute VZV infection over a period of 11 months (Figure 5). However, comparison of the CVs demonstrated that avidity calculations using a fixed x were less influenced by the chosen dilution than avidity indices based on the slope (data not shown).

Further dilution series of CSF and corresponding serum were tested with the CSF avidity assay in order to confirm that the influence of the IgG concentration on the avidity indices derived from the virtual absorbance ratio was minimal. Table 2 shows that the CVs with this method were equally low with CSF and serum. Results obtained with the software "Avidity 1.2" and results based on the absorbance ratios of single dilutions are presented for comparison. Although for two samples the CVs with "Avidity 1.2" were slightly lower than with the new method (CSF of V2.1 and CSF of V9.2), for the others the new method gave lower CVs. Avidity values derived from absorbance ratios resulted in the highest CVs for all samples.

## Discussion

We established a VZV avidity assay that is suitable for comparative evaluation of antibody avidity in serum and CSF samples. In order to achieve this aim several modifications of a serum VZV avidity assay were necessary. Though there have been a few studies on the use of avidity assays for VZV IgG antibodies [16-18], no standard method or commercial assays exist for the determination of VZV IgG avidity. Therefore, we first established conditions for a VZV IgG avidity assay that can be used for the differentiation of acute and past VZV infection. The serum assay was highly sensitive and specific for the diagnosis and exclusion of primary VZV infections. However, diagnostic applications of VZV IgG avidity determinations with serum samples are rare, because the diagnosis of acute or reactivated VZV infection is usually made clinically or by virus detection methods.

Urea hydrogen peroxide appeared to be superior to urea in the optimization experiments for the VZV avidity assay and presents a novel option for a denaturing agent in avidity measurements. The denaturing effect of one washing step with a 4 M solution of urea hydrogen peroxide was more pronounced than that of three washing steps with a 5 M urea solution. Thus, the denaturing potential of urea hydrogen peroxide appears to be greater than that of urea. Furthermore, because of better solubility, handling of solutions of urea hydrogen peroxide is easier than handling solutions of urea in high molarity. Nevertheless, the optimal denaturing agent may vary between different antigens necessitating careful evaluation of denaturing conditions for each avidity assay.

Under normal conditions, virus specific IgG is present in the CSF in very low concentrations. With an intact blood-CSF-barrier, the concentration gradient between serum and CSF is 200:1 to 1000:1. Thus, the usual serological methods for antibody quantification in serum are not sensitive enough to routinely detect specific IgG in CSF. Therefore, we modified the VZV IgG EIA to increase its sensitivity. This modification made it possible to detect and quantify VZV IgG in CSF samples from virtually all patients with measurable VZV IgG in serum. Corresponding serum samples were always run in parallel with CSF in the same assay. The CSF and serum dilutions were chosen individually for each sample pair in order to achieve similar absorbance values in the modified EIA. This assay formed the basis for avidity determination of CSF and the corresponding serum.

For meaningful comparison of avidity values in serum and CSF, it is necessary to apply a calculation method that is independent of the concentration of specific IgG in the samples examined. One-point determination represents the simplest method for avidity index calculation, but such methods did not yield satisfactory results in this respect. The avidity technique based on end-point-titration is not influenced by the IgG concentration and is considered the reference method for avidity determination [1,7]. However, it is relatively laborious and reagent consuming, requiring two distinct dilution series for the assays with and without protein denaturant. Therefore, we attempted to use the logistic approach based on end-point-titration, with fewer dilutions per sample [15]. This method works well in distinction between acute and past infections from samples of serum. However, under the conditions of the CSF avidity assay we found that the results of the logistic model were not uniformly independent of the sample working dilutions, albeit without straightforward dose-dependence of specific IgG. The reason for this limitation with our assay may lie in its modifications to increase sensitivity. Possibly, the curve fitting obtained with the logistic model would have to be adjusted in order to account for the special conditions of the CSF avidity assay.

Searching further for a simple calculation method of avidity indices independent of IgG concentration and sample dilutions, we observed a linear relationship between the absorbance values of the assays without and with denaturing washing conditions. After performing an avidity assay of two dilutions of a given sample, this relationship can be exploited to calculate a linear equation from the two pairs of absorbance values (with and without denaturation). The only requirement is that all absorbance values should fall in the linear range of the EIA. Once for a given sample the linear equation is experimentally determined, the equation can be used for calculation of the virtual absorbance value with denaturation (abs_denat_) for any virtual absorbance value without denaturation (abs_ref_). The avidity index is then calculated as the ratio of the two virtual absorbance values (abs_denat_/abs_ref_). Thus, if a fixed abs_ref _is chosen for all samples to be compared, the avidity index becomes independent of the concentration of specific IgG in the samples. These theoretical considerations have been confirmed by the results obtained from dilution series in this study. It will be interesting to test the general applicability of this method for standard avidity assays by comparison with single-point and end-point avidity determinations.

## Conclusions

In summary, we have described several modifications of existing avidity techniques that have the potential to broaden the use of avidity assays in diagnosis and research. Urea hydrogen peroxide is a novel denaturing agent, which appears to be advantageous compared to urea in terms of handling conditions. The new calculation method of avidity indices based on a linear equation with fixed reference absorbance values is cost-effective and simple and has the potential to substitute other avidity calculation procedures. The CSF avidity assay in combination with the new calculation method allows for determination of the avidity of VZV IgG in corresponding serum and CSF samples with high precision and independent of the VZV IgG concentration. These features are necessary in order to study the avidity maturation in serum and CSF in patients with intrathecal synthesis of VZV-specific IgG antibodies.

## Competing interests

BW has received research and travel grants from Dade Behring. KH is employed by an organization (The Helsinki University Hospital Laboratory, HUSLAB) using the Avidity 1.2 software in infectious-disease diagnosis, and is a shareholder of an SME (Headman Ltd.) with commercial interest in it.

## Authors' contributions

RHK, JS and FT carried out the immunoassays and participated in the design of the study. RHK carried out the avidity index calculations. WZ participated in establishing the standard VZV IgG avidity assay. KH provided the software "Avidity 1.2" and participated in the data analysis. BW conceived of and coordinated the study and drafted the manuscript. All authors contributed to the final editing of the manuscript.

## Pre-publication history

The pre-publication history for this paper can be accessed here:



## Figures and Tables

**Table 1:** Comparison of the absorbance values with different denaturing washing conditions in the VZV IgG avidity assay.

VZV infection status	3 × 5 min 5 M urea			1 × 3 min 4 M urea H_2_O_2_		
	Abs (ref)^1^	Abs (denat)^2^	Avidity	Abs (ref)	Abs (denat)	Avidity
		
acute	2.221	1.208	54.4%	2.359	0.546	23.2%
acute	1.829	0.676	37.0%	1.666	0.300	18.0%
acute	1.982	0.457	23.1%	1.869	0.251	13.4%
past	1.487	1.387	93.3%	1.448	0.850	58.7%
past	1.190	0.830	69.7%	1.072	0.580	54.1%
past	2.020	1.671	82.7%	1.929	0.984	51.0%

^1^absorbance value in EIA without denaturing washing step

^2^absorbance value in EIA with denaturing washing step

**Table 2:** Comparison of avidity values from serum and CSF pairs obtained with different calculation methods from different dilutions (absorbance ratios) or different pairs of dilutions (new method and "Avidity 1.2"). For each method, mean values, standard deviations (SD) and coefficients of variation (CV) are presented.

	V2.1		V9.2		M22	
	Serum	CSF	Serum	CSF	Serum	CSF
			
**absorbance ratios**						
Mean (%)	50.2	54.0	59.9	66.4	34.7	38.6
SD (%)	5.6	5.4	7.8	8.5	4.1	6.1
CV (%)	11.1	10.0	13.0	12.8	12.0	15.9
**Avidity 1.2**						
Mean (%)	37.6	45.4	50.5	56.5	23.2	31.9
SD (%)	3.7	3.0	3.0	4.6	2.3	3.6
CV (%)	9.9	6.6	5.9	8.1	9.9	11.4
**new method**						
Mean (%)	49.8	58.4	65.2	72.6	36.5	45.1
SD (%)	2.4	4.1	1.4	7.5	2.0	0.9
CV (%)	4.9	7.1	2.1	10.3	5.6	1.9

**Table 3:** Calculation of avidity indices from dilution series of representative samples with the software "Avidity 1.2" and with the new calculation method described in this paper.

(a) Raw data of dilution series						
	Serum F19.3		Serum P28		Serum V9.2	
Relative dilution^1^	Abs (ref)^2^	Abs (denat)^3^	Abs (ref)	Abs (denat)	Abs (ref)	Abs (denat)
			
1:1	2.765	0.925	2.668	1.600	2.936	1.948
1:2	1.962	0.626	1.833	1.144	1.943	1.268
1:4	1.428	0.356	1.300	0.727	1.269	0.782
1:8	0.831	0.190	0.824	0.441	0.778	0.463
1:16	0.592	0.106	0.480	0.252	0.409	0.192

(b) Avidity indices obtained from different combinations of dilutions with arithmetic mean, standard deviation (SD) and coefficient of variation (CV)						

	Serum F19.3		Serum P28		Serum V9.2	
Dilutions used for calculation	Avidity 1.2 (%)	new method (%)	Avidity 1.2 (%)	new method (%)	Avidity 1.2 (%)	new method (%)
			
1:1 & 1:2	9.5	32.0	33.4	61.8	46.1	65.4
1:2 & 1:4	15.2	32.3	40.4	63.7	49.2	65.5
1:4 & 1:8	18.0	25.8	41.5	57.4	50.0	62.9
1:8 & 1:16	11.7	30.0	44.6	54.4	56.6	68.0
1:1 & 1:4	11.6	30.0	35.5	58.7	48.2	64.7
1:2 & 1:8	17.3	32.0	41.8	63.0	50.1	65.4
1:4 & 1:16	13.5	26.4	43.5	56.6	50.2	64.2
1:1 & 1:8	14.7	31.7	41.3	59.0	52.8	65.2
1:2 & 1:16	15.0	32.0	42.0	62.7	51.3	65.4
Mean	14.1	30.2	40.4	59.7	50.5	65.2
SD	2.8	2.5	3.7	3.3	3.0	1.4
CV	19.7	8.4	9.0	5.5	5.9	2.1

^1^Relative dilution: the starting dilutions were 1:1512 (F19.3), 1:144 (P28), 1:399168 (V9.2)

^2^absorbance value in EIA without denaturing washing step

^3^absorbance value in EIA with denaturing washing step
